# Effects of Welfare Reform on Positive Health and Social Behaviors of Adolescents

**DOI:** 10.3390/children10020260

**Published:** 2023-01-31

**Authors:** Nancy E. Reichman, Hope Corman, Dhaval Dave, Ariel Kalil, Ofira Schwartz-Soicher

**Affiliations:** 1 Department of Pediatrics, Robert Wood Johnson Medical School, Rutgers University, New Brunswick, NJ 08903, USA; 2 Department of Economics, Rider University, Lawrenceville, NJ 08648, USA; 3 National Bureau of Economic Research, Cambridge, MA 02138, USA; 4 Department of Economics, Bentley University, Waltham, MA 02452, USA; 5 IZA Institute of Labor Economics, 53113 Bonn, Germany; 6 Harris School of Public Policy, University of Chicago, Chicago, IL 60637, USA; 7 Donald E. Stokes Library, Princeton University, Princeton, NJ 08544, USA

**Keywords:** welfare reform, adolescents, health behaviors, school behaviors

## Abstract

This paper explores a missing link in the literature on welfare reform in the U.S.—the effects on positive health and social behaviors of adolescents, who represent the next generation of potential welfare recipients. Previous research on welfare reform and adolescents has focused almost exclusively on negative behaviors and found that welfare reform led to decreases in high school dropout and teenage fertility among girls, but increases in delinquent behaviors and substance use, particularly among boys. Using nationally representative data on American high school students in 1991–2006 and a quasi-experimental research design, we estimated the effects of welfare reform implementation on eating breakfast, regular fruit/vegetable consumption, regular exercise, adequate sleep, time spent on homework, completion of assignments, participation in community activities or volunteering, participation in school athletics, participation in other school activities, and religious service attendance. We found no robust evidence that welfare reform affected any of these adolescent behaviors. In concert with the past research on welfare reform in the U.S. and adolescents, the findings do not support the implicit assumption underlying welfare reform that strong maternal work incentives would increase responsible behavior in the next generation and suggest that welfare reform had overall adverse effects on boys, who have been falling behind girls in terms of high school completion for decades.

## 1. Introduction

The U.S. 1996 Personal Responsibility and Work Opportunity Reconciliation Act (PRWORA) and similar policies that preceded it (collectively referred to as welfare reform), aimed to reduce dependence on cash assistance by encouraging maternal employment through work requirements and time limits, encouraging marriage, and strengthening child support. An implicit assumption behind the reforms was that a work- and responsibility-focused regime would not only encourage employment and other prosocial behaviors of mothers, but that such positive behaviors would translate to the next generation and disrupt a presumed intergenerational transmission of welfare dependence. 

The reforms were successful in that average monthly family welfare caseloads in the United States declined by 80% between 1994 and 2019 [1,2] and employment of low-skilled women increased by as much as 27% [3,4]. However, the reforms had weak to no effects on marriage [4]. Other studies found that welfare reform led to declines in women’s substance abuse [5,6] and crime [7], and increases in women’s civic participation in the form of voting [8]. Overall, findings support the assumption underlying welfare reform that strong work incentives would encourage responsible behavior.

Fewer studies have investigated how the new regime has affected behaviors of the next generation of children (those old enough to make autonomous decisions), which is an area of inquiry that could potentially provide some early life course evidence on the integrity of the intergenerational “culture of poverty” argument. PRWORA led to decreased high school dropout of girls [9] and teen fertility [10], but part of the effect was through requirements for mothers <18 (a group of adolescents who were directly affected through the legislation rather than indirectly through their mothers) to participate in education or training and live with a parent/guardian. Syntheses of findings from pre-PRWORA welfare experiments in specific geographic areas found that the programs were associated with worse school performance, more grade repetition, and more special education of adolescents, but no differences in drop out, suspension from school, school completion, or childbearing, and few gender differences in outcomes overall [11]. Few gender differences were explored and not all of these outcomes are clear measures of adolescents’ responsible or irresponsible behavior (e.g., school completion would be, but grade repetition could reflect maternal proactivity rather than a child’s lack of effort).

Three recent studies exploited differences in the full implementation of welfare reform across states and over time to identify causal effects of welfare reform on teen arrests and various types of adolescent delinquent behavior, and together, found that welfare reform led to few improvements in behaviors of adolescent girls and had detrimental effects on behaviors of adolescent boys [12,13,14]. As such, these findings do not support the argument that welfare reform would encourage responsible behavior in the next generation. However, as far as we know, no previous studies have evaluated the impact of welfare reform on positive indicators of productivity and responsibility of adolescents in the next generation. That is, studies have not addressed the possibility that there were potentially offsetting favorable effects.

In this paper, we address this gap by investigating overall and gender-specific effects of welfare reform on a range of positive health and social behaviors of high school students that have been associated with long-term health and/or socioeconomic outcomes. These include measures of healthy eating, regular exercise, adequate sleep, doing homework, volunteering, participating in school athletics and other extracurricular activities, and religious attendance. These behaviors can change within a relatively short time period and represent early observable consequences of the reforms for the next generation in a particularly important period of development when decisions can have long-term consequences for future economic success and health [15]. Welfare reform could improve teens’ behaviors through increases in income via maternal employment (e.g., allowing for increased investments in health care, education, or after school activities), by leading mothers to model socially desirable behavior, and/or by changing youths’ expectations about welfare as a long-term option and thereby leading them to focus more on school and community activities as investments in human and social capital. However, welfare reform could also lead to a net increase in constraints (e.g., if increases in income do not offset transportation and childcare expenses, or by decreasing time available for supervision), which could lead to undesirable effects on teens’ behaviors.

## 2. Materials and methods

### 2.1. Data

We used restricted data from the 10th and 12th grade surveys of Monitoring the Future (MTF), an annual nationally representative survey of high school students, for the years 1991–2006 [16]. 1991 was the year the MTF began surveying 10th graders and it preceded welfare reform in all states. Using 2006 as the endpoint allowed all states to have fully implemented welfare reform but avoided conflating our results with the effects of the Great Recession that began in the last quarter of 2007. The MTF is administered at over 400 public and private schools and provides representative samples of students in 8th, 10th, and 12th grades. Between 13,000 and 19,000 students are surveyed each year in each of the three grades. We limited our sample to high school students (grades 10 and 12) who were minors (<18 years old). We focused on 10th and 12th graders who are likely more autonomous decision-makers than 8^th^ graders. We focused on the teens whose mothers had a high school education or less and were unmarried, a group we refer to as our “target” group. Low-educated unmarried mothers are at high risk for welfare participation [17], and therefore potentially impacted the most by welfare reform in terms of their employment, income, and other household resources. Thus, we would expect the largest welfare reform-induced behavioral effects on the children of mothers in this group, if there were any. In some analyses, we also used a comparison group consisting of teens whose mothers had a high school education or less but were married—a group similar in many ways to our target group, but less likely to be eligible for welfare.

### 2.2. Measures

#### 2.2.1. Outcomes

We focus on health, school, and social behaviors that have been shown to positively affect health and socioeconomic status, which themselves are strongly intertwined [18]: Frequently eating breakfast, regular consumption of fruits and vegetables, regular exercise, adequate sleep, time spent on homework, completion of assignments, volunteering, participation in athletics and other extracurricular activities, and regular attendance at religious services. Some examples of the importance of these outcomes include: A recent review article finding that eating breakfast has positive associations with children’s cognitive performance and academic achievement [19]. Numerous studies finding life-long health benefits of adolescent consumption of fruits and vegetables [20], that regular physical activity in adolescents confers lifelong cardiovascular benefits [21], and that adolescents who get adequate sleep have better metabolic health [22]. Studies finding that more time spent on homework results in higher grades in school and an increased likelihood of attending college [23] and that community engagement in adolescence, such as participation in school clubs/organizations, participation in community service organizations, and volunteering for social causes, has been linked to student achievement and civic participation, educational attainment, and lower arrest rates in emerging adulthood [24,25,26]. Research finding that religious attendance during adolescence has been linked to decreased likelihood of substance abuse [27].

In the MTF survey, the students were asked about: (1) The frequency with which they engaged in eating breakfast, eating fruits and vegetables, exercising, and sleeping for 7+ hours, with response categories ranging from “never” to “every day”. For each, we created a binary variable for “most days”, “nearly every day” or “every day” versus “never”, “seldom” or “sometimes”. (2) The average number of hours per week spent on homework both in and out of school. We created a binary variable for 10 or more hours versus fewer (categories were 0, 1–4, 5–9, 10–14, 15–19, 20–24, 25+ h). (3) How often they failed to hand in assignments. We created a binary variable for never or seldom versus sometimes or often. (4) The frequency with which they participated in community activities or did volunteer work. We created a binary variable for almost every day, at least weekly, or once or twice a month versus a few times a year or never. (5) The extent to which they engaged in school athletics and other school activities (newspaper or yearbook, music or other performing arts, other clubs or activities). For each, we created a binary measure for considerable or great extent (versus not at all, slightly or moderately). (6) How frequently they attended religious services. We created a binary variable for reports of once or twice a month or weekly versus rarely or never.

#### 2.2.2. Welfare Reform

PRWORA ended entitlement to welfare under Aid to Families with Dependent Children (AFDC) and replaced AFDC with Temporary Assistance for Needy Families (TANF) block grants to states. Features of the legislation included time limits on cash assistance, work requirements as a condition for receiving benefits, and increased state latitude in establishing eligibility and program rules. Although welfare reform is often dated to the PRWORA legislation, reforms started taking place in the early 1990s in the form of “welfare waivers” that allowed states to conduct experimental changes to their AFDC programs. Waivers were implemented in most states by the time the federal PRWORA was enacted in 1996. Many policies and features of PRWORA, such as work requirements and time-limited welfare receipt, were integral parts of these earlier programs. Major statewide waivers were introduced in 29 states over 53 months, and TANF was implemented in all states over a period of 17 months [28]. Considering both waivers and TANF, states implemented any welfare reform over a period of 64 months, from October 1992 through January 1998.

For our analyses, we exploited differences in the timing of both AFDC waivers and TANF implementation across states. For waivers, we considered whether a state had a statewide AFDC waiver in place in each month/year. For TANF, we considered whether the state had implemented TANF post-PRWORA in each month/year. In most specifications, we included a single indicator for any welfare reform (AFDC waiver or TANF). In supplementary models, we used separate indicators for AFDC and TANF.

We matched the timing of each phase of welfare reform to the teens’ surveys based on maternal state of residence and month/year of interview. A teen was considered exposed to welfare reform if the mother resided in a state in which welfare reform had been in effect for at least 12 months (i.e., welfare reform had been implemented at least 12 months before the month of interview). The 1-year lag addressed the retrospective nature of the outcomes, many of which capture participation over the past year, and also allowed for time for maternal exposure to welfare reform to affect children’s behaviors.

#### 2.2.3. Covariates

Individual-level covariates included the child’s age, grade in school, sex (in pooled models), and race/ethnicity (white, black, or other, with the last category including Hispanics), and the mother’s education and marital status. State/year covariates included unemployment rates, poverty rates, personal income per capita, Earned Income Tax Credit (EITC) rates, whether the state had a refundable EITC, minimum wage, number of Medicaid beneficiaries, number of National School Lunch and School Breakfast Program participants, and population. Models also included lagged economic/welfare conditions (one, two, and three-year lags of the unemployment rate, poverty rate, state personal income per capita, welfare caseloads), and indicators for state, year and month.

### 2.3. Analyses

We capitalized on quasi-experimental variation in the timing of welfare reform implementation across states by estimating difference-in-differences (DD) and differences-in-differences-in differences (DDD) Ordinary Least Squares models (see Appendix A). We estimated models that pooled both genders (with a control for gender) and gender-specific models. We also conducted a number of sensitivity checks.

## 3. Results

Adolescents in the target group were somewhat less likely than those in the comparison group to engage in the behaviors examined (Table 1). The major differences between the target and comparison groups were that adolescents in the target group were more likely to be Black and have mothers without a high school degree. Otherwise, the two groups were similar across all characteristics.

**Figure 1:**
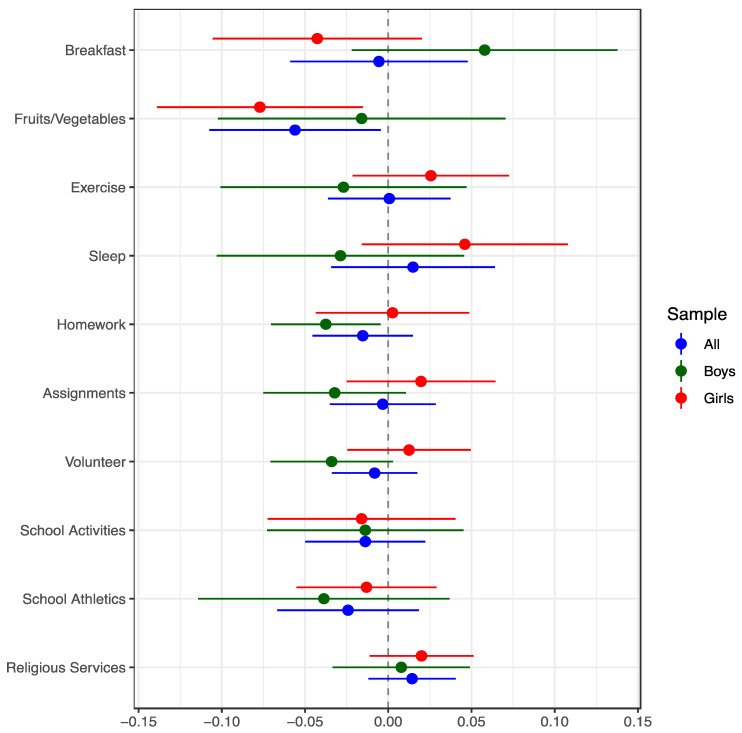
Ordinary Least Squares Difference-in-Differences (DD) Estimates of the Effects of Welfare Reform on Positive Adolescent Health and Social Behaviors, United States. Notes: The sample consisted of 10th and 12th graders (<age 18 years) whose mothers had a high school education or less and were unmarried. DD coefficients of the welfare reform indicator (Welfare, from Equation (A1) in the Appendix A) are shown, with 95% confidence intervals. The models controlled for the characteristics listed in Table 1, state/year covariates (unemployment rate, poverty rate, personal income per capita, Earned Income Tax Credit (EITC) rate, refundable EITC, minimum wage, # Medicaid beneficiaries, #s of National School Lunch and School Breakfast Program participants, and population), lagged economic/welfare conditions (one, two, and three-year lags of the unemployment rate, poverty rate, state personal income per capita, and welfare caseloads) and indicators for state, year and month. Sample sizes ranged from 7358–24,272 (pooled genders), 2992–9704 (boys), and 4366–14,658 (girls).

In DD models, welfare reform was associated with a 4.3–7.7 percentage point decrease (9.6–16.7% relative to the mean) in frequent breakfast and fruit/vegetable consumption for girls, whereas the estimates were positive (for breakfast) or weakly negative (for fruits and vegetables) for boys (Figure 1). The opposite is true for the other behavioral outcomes (i.e., the coefficients are generally more negative or less positive for boys than girls). However, almost all estimates are not statistically distinguishable from zero.

**Figure 2:**
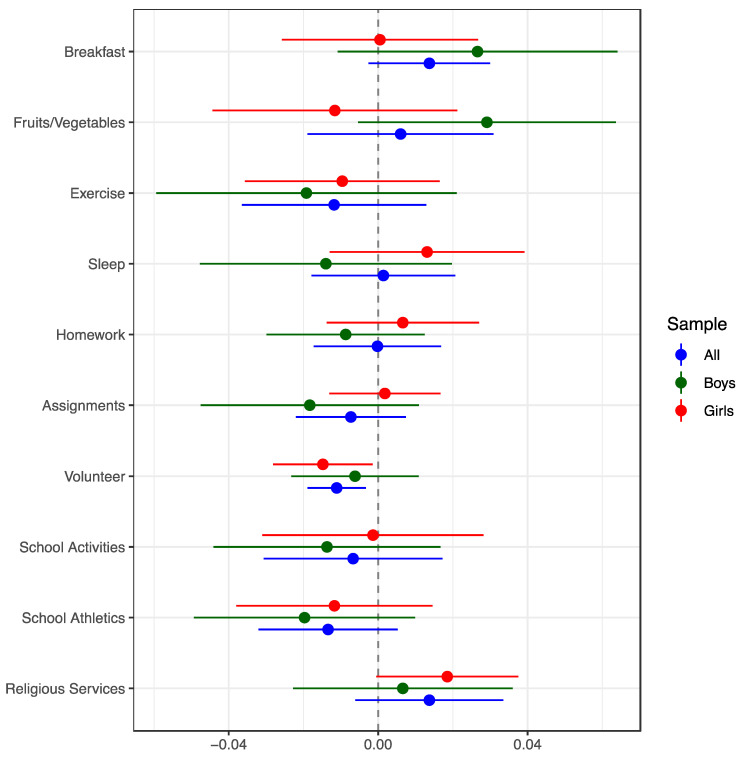
Ordinary Least Squares Difference-in-Difference-in-Differences (DDD) Estimates of the Effects of Welfare Reform on Positive Adolescent Health and Social Behaviors, United States. Notes: The sample consisted of 10th and 12th graders (<age 18 years) whose mothers had a high school education or less. The target group included the subset whose mothers were unmarried, and the comparison group included those whose mothers were married. DDD coefficients of the interaction between the welfare reform and target group indicators (*Π*_2_, from Equation (A2) in the Appendix A) are shown, with 95% confidence intervals. The models controlled for the main effects of welfare reform and the characteristics listed in the notes to Figure 1. Sample sizes ranged from 35,430–109,607 (pooled genders), 15,637–48,242 (boys), and 19,793–61,365 (girls).

The DDD estimates are generally similar to the DD estimates (Figure 2). An exception is for volunteering, for which the DDD estimate suggests a significant welfare reform-associated decrease for girls (1.5 percentage points) but not boys. Overall, the DD and DDD analyses strongly suggest that welfare reform had little impact on adolescents’ positive behaviors.

We implemented several specification checks to assess sensitivity of the estimates. We decomposed the composite effect of welfare reform into AFDC waivers and TANF implementation and found no differential effects by phase of welfare reform. We compared models that included and excluded the state covariates and found that the effects of state welfare reform implementation did not appear to be associated with other state policies. We also found that the results were insensitive to adding controls for state-specific linear trends, which allowed all states (including early- and late-reform states) to have differential systematic trends over the entire sample period; controlling for zip code and school; using alternative cutoffs for the binary outcome variables; and using an alternate comparison group in DDD models that consisted of children with unmarried mothers who had more than a high school education.

## 4. Discussion

Despite large sample sizes and the consideration of a wide range of relevant behaviors, we did not find evidence that welfare reform led to changes in adolescents’ positive health and social behaviors. The few significant associations were not robust across model specifications. These findings add to an emerging literature on the effects of welfare reform on adolescent behavioral outcomes beyond the effects on high school dropout and teen fertility of girls.

Using FBI arrest data, Corman et al. (2017a) found that welfare reform had no statistically significant effect on teen drug arrests, although most estimates were positive and suggestive of a small (3%) increase in arrests [12]. Additionally, using FBI arrest data, Corman et al. (2017b) found that welfare reform led to reduced youth arrests for minor crimes (e.g., liquor law violations, disorderly conduct, loitering), by 7–9%, with similar estimates for boys and girls, but did not affect youth arrests for serious crimes (e.g., violent crimes, such as murder and assault, property crimes, such as burglary and larceny) [13]. The authors acknowledged that the results could reflect welfare reform-induced decreases in minor crime but could also reflect decreases in the probability of getting caught, and that it was not possible to disentangle those two potential effects using FBI arrest data.

Using individual-level MTF data, Dave et al. (2021) estimated gender-specific effects of welfare reform on a range of behaviors of high school-aged youth, such as skipping school, getting into fights, damaging property, stealing, hurting others, and various types of substance use. For boys, welfare reform led to a significant increase in skipping school, damaging property, fighting, stealing, and hurting others, with an average effect of 0.08 standard deviations [14]. For girls, there were no systematic effects of welfare reform on any of these behaviors, other than a small welfare reform-associated increase in skipping school. For both boys and girls, welfare reform led to significant increases in marijuana, cigarette, and other illicit drug use, particularly for boys (~0.06 standard deviations vs. ~0.04 standard deviations for girls). These findings were consistent with those of Corman et al. (2017a) and suggest that the findings of Corman et al. (2017b) reflected welfare reform-induced decreases in probability of arrest rather than decreases in crime and underscore the importance of measuring behaviors directly and considering gender-specific effects.

In this study, we found that welfare reform did not lead to improvements in positive social and health behaviors that could potentially have offset the strong detrimental effects of welfare reform on adolescent delinquent behaviors, suggesting that the net effects of welfare reform on adolescents were unfavorable, particularly for boys. In addition, the findings from this study in concert with those from the previous literature do not support the implicit assumption behind welfare reform that strong maternal work incentives would increase responsible behavior in the next generation. Overall, the findings on intergenerational effects of welfare reform to date do not bode well for the well-being of the next generation—in particular for boys, who have been falling behind girls in terms of high school completion for decades [29]. That said, the findings pertain to a relatively early stage in the life course and it is important to investigate longer-term outcomes—e.g., a recent study found that welfare led to reductions in food insecurity in the next generation of households that were much stronger for women than men [30]. It is also important to investigate the effects of welfare reform on parenting inputs, home environments, and other factors that might explain the adverse effects of welfare reform on the behaviors of teens despite favorable effects on many behaviors of mothers.

## Figures and Tables

**Table 1:** Outcomes and Sample Characteristics.

	Sample		
Measures	All Teenage children with low-educated mothers	Target Group: Teenage children with low-educated unmarried mothers	Comparison Group: Teenage children with low-educated married mothers
Outcomes			
Breakfast	0.45	0.39	0.46
Fruits/vegetables	0.46	0.40	0.48
Exercise	0.58	0.52	0.59
Sleep	0.65	0.61	0.66
Homework	0.23	0.20	0.24
Assignments	0.54	0.49	0.56
Volunteer	0.24	0.23	0.25
School activities	0.41	0.38	0.42
School athletics	0.37	0.32	0.38
Religious services	0.49	0.43	0.50
Characteristics			
Child:			
Age (years)	16.0	16.1	16.0
Grade 10	0.71	0.71	0.70
Grade 12	0.30	0.29	0.30
Male	0.45	0.41	0.46
Black	0.11	0.27	0.07
Other non-White	0.23	0.23	0.23
Mother:			
Grade school	0.07	0.07	0.07
Some high school	0.21	0.27	0.20
High school graduate	0.72	0.67	0.74
Married	0.78	0.00	1.00
Sample size	111,031	24,649	86,382

Notes: Weighted column proportions or means (for age) are reported. For some outcomes, samples sizes are lower than those indicated due to sampling design or missing information.

## Data Availability

This study used retrospectively collected de-identified data (Monitoring the Future Restricted-Use Cross-Sectional Datasets) that are publicly available through a restricted use data contract. The data were obtained from: https://monitoringthefuture.org/results/data-products/.

## Appendix A

We started with the following reduced-form difference-in-differences (DD) setup that directly links a teen’s behaviors (*B*_imst_) to their exposure to welfare reform (*Welfare*):

B_imst_|_Target_ = Π_1_ (Welfare_st-12_) + X_imst_ β + V_mst_ λ + Z_st_ δ + State_s_ Ω + Time_t_ Ψ + ε_imst_ (A1)

Subscripts denote the *ith* child born to mother *m* residing in state, *s,* and observed at time, *t*. *Welfare* is measured by an indicator for whether a given state had in place a major AFCD waiver or had implemented TANF for at least 12 months.

We controlled for vectors of child characteristics (*X*_imst_) and maternal characteristics (*V*_mst_). To account for potentially confounding policy shifts, a rich set of time-varying state factors (*Z*_st_), was included. Vector *Z* also contained lagged state-level economic conditions and welfare caseloads, to account for the potential endogeneity of the timing of policy adoption, which may be linked to the state’s recent economy and welfare caseloads. Models further included *State* and *Time* (month/year of interview) fixed effects, which control for fixed state heterogeneity, national trends, and seasonal variations in youth outcomes.

Equation (A1) was estimated for our population of interest, which was children born to women at risk of relying on public assistance—those who had a high school education or less and were unmarried. However, while the DD specification included a rich set of time-varying state controls, the possibility of omitted variables remained. This was addressed within a difference-in-difference-in-differences (DDD) setup (Equation (A2) below) that used a comparison group—individuals similar in many ways to the target group but unlikely to be affected by welfare policy—to net out residual unobserved influences on outcomes that may be correlated with welfare reform. We followed the literature and utilized children of low-educated mothers who were married as a comparison group. As marriage generally precludes eligibility for cash assistance, married mothers are at much lower risk of welfare receipt than those who are unmarried and thus far less likely to be affected by welfare reform.

B_imst_ = α + Π_2_ (Welfare_st-12_ * Target_mst_) + Π_3_ Welfare_st-12_ + X_imst_ β + V_mst_ λ + Z_st_ δ + State_s_ Ω + Time_t_ Ψ + ε_imst_ (A2)

*Target* represents a dichotomous indicator equal to 1 if the teen is in the target group (had a low-educated unmarried mother, and thus was at risk of being on welfare) and 0 if he/she is in the comparison group (had a mother who is not at risk of being on welfare). The estimate of interest is Π_2_, the DDD coefficient of the interaction between the *Welfare* and the *Target* group indicators, which represents the reduced-form effect of exposure to welfare reform on the teen’s behavior operating through any and all reinforcing and/or offsetting channels. We adjusted standard errors for arbitrary correlation in the error term (ε) across and within individuals in a given state.
